# Hyaluronan-Based Hybrid Systems as Growth Factor Carriers in the Treatment of Chronic Wounds

**DOI:** 10.3390/ijms262210871

**Published:** 2025-11-09

**Authors:** Aneta Ostróżka-Cieślik, Archana Tanwar, Monika Michalak

**Affiliations:** 1 Department of Pharmaceutical Technology, Faculty of Pharmaceutical Sciences in Sosnowiec, Medical University of Silesia, 41-200 Sosnowiec, Poland; 2 Department of Chemistry, Fergusson College (Autonomous), Pune 411004, India; archana.tanwar@fergusson.edu; 3 Department of Pharmaceutical Sciences, Medical College, Jan Kochanowski University, 25-317 Kielce, Poland; monika.michalak@ujk.edu.pl

**Keywords:** hyaluronan, growth factors, hybrid polymeric hydrogel, hybrid polymeric system, wound healing, chronic wounds

## Abstract

The treatment of wounds, most of which are complications of chronic diseases, poses a significant clinical challenge. Hybrid systems based on hyaluronic acid containing growth factors are a promising prospect for the treatment of chronic wounds. Hyaluronic acid supports fibroblast proliferation, migration, and adhesion to the wound site, and stimulates collagen production. Growth factors (GF), such as epidermal growth factor (EGF), fibroblast growth factor (FGF), and vascular endothelial growth factor (VEGF), influence the normal proliferation and migration of keratinocytes and fibroblasts. This review aims to summarise the current state of knowledge regarding their therapeutic potential. Google Scholar, Web of Science, and Medline (PubMed) databases were searched. Eighteen studies, including basic, preclinical, and clinical studies, were included in the review. The studies confirm the therapeutic potential of the developed formulations. Collagen/hyaluronic acid and alginate/hyaluronic acid systems are biocompatible and biodegradable matrices that provide a moist wound environment, which promotes cell migration and proliferation. EGF stimulates the proliferation and migration of keratinocytes, which accelerates re-epithelialisation. bFGF supports angiogenesis by stimulating the proliferation and migration of vascular endothelial cells. The effect of these actions indirectly leads to increased production of VEGF and HGF cytokines, which support the formation of granulation tissue. The VEGF-containing dressing stimulated vascularisation and the production of collagen type-1 and fibronectin. Only one clinical study conducted in this field indicates the need for further research in this area.

## 1. Introduction

Hybrid hydrogels were developed to address the limitations of native hydrogels. The versatile properties of hybrid hydrogels (including increased strength, drug release efficiency, and therapeutic efficacy) are particularly valued in the biomedical sector as a promising tool for tissue regeneration and wound healing [1].

One of the definitions describes hybrid hydrogels as complexes formed by combining various polymers and/or integrating nanoparticles. Hybrid hydrogel superstructures contain biomaterials, nanomaterials, and nanobiocomposites in their polymer network while also containing one or more active substances such as small molecules, peptides, proteins, and nucleic acids. These modern systems combine the characteristic features of conventional synthetic hydrogels (such as hydrophilicity and exceptionally high water transport capacity) with the unique features of nanomaterials and bionanocomposites (very small sizes and unique optical, electrical, and mechanical properties [1,2,3]. Biomaterial-based hybrid hydrogels consist of synthetic polymers (i.e., polyethylene glycol (PEG), polyvinyl alcohol (PVA), and polyacrylamide) and natural polymers (i.e., collagen, hyaluronic acid, gelatin, chitosan, and alginate) [1,2]. They are of great interest to researchers due to their ability to mimic the extracellular matrix, as well as their biocompatibility, biodegradability, and environmental friendliness. In addition, hybrid hydrogels should exhibit the ability to gel under physiological conditions, appropriate porosity, controlled swelling, and the capacity for functionalization to incorporate bioactive molecules [1,2,4]. Advanced material systems exhibit improved physical, chemical, and mechanical properties and demonstrate greater potential and applicability in tissue engineering, drug delivery, and wound healing. They are remarkable, multifunctional, intelligent hydrogels for the healthcare sector [1,2].

It is estimated that 4% of the global population suffers from chronic wounds, and this percentage is growing as the number of elderly people in the world increases [5]. The treatment of chronic wounds generates high costs associated with long-term treatment, hospitalisation, and social expenditure, which poses a significant challenge for healthcare systems and the fields of economics and epidemiology [6,7]. Chronic wounds are defined as skin lesions that heal abnormally and remain open for more than a month [8]. They are a significant clinical problem and are the result of complications from chronic diseases such as diabetes, obesity, and cardiovascular disease [6,8]. The most common types of hard-to-heal wounds include diabetic foot syndrome, venous and arterial ulcers, and pressure sores [9]. In these wounds, normal healing processes are disrupted, which can result in prolonged inflammation, infection, and tissue necrosis. Persistent inflammation inhibits normal proliferation and angiogenesis processes through the overproduction of pro-inflammatory cytokines and proteolytic enzymes, local hypoxia, the presence of bacterial biofilm, and impaired angiogenesis, resulting in weak and damage-prone scar tissue [9]. In addition, the course of chronic wounds is often complicated by comorbidities [5,6,7].

## 2. Hyaluronic Acid—Properties and Biomedical Potential

Hyaluronic acid (HA) is a naturally occurring linear polysaccharide that belongs to the glycosaminoglycan family. It is a non-sulfated glycosaminoglycan predominantly found in all vertebrates, including humans. In the human body, HA is synthesized by three transmembrane glycosyltransferase isoenzymes named hyaluronan synthases, HAS: HAS1, HAS2, and HAS3. Hyaluronic acid degradation occurs via two different mechanisms. One involves enzymes (hyaluronidase (HYAL)), and the other is determined by oxidative damage caused by reactive oxygen species (ROS) [10]. In humans, it is prevalent in the skin and the extracellular membrane, primarily serving as a lubricant because of its hydrophilic nature, and aids in maintaining skin hydration and resilience [11,12,13]. It is estimated that more than half of the HA in the human body is located in the skin, of which the dermis contains approximately 200–500 μg/mL and the epidermis 100 μg/mL of HA [14]. HA determines the elasticity, moisture content, and desired structure of the skin. Furthermore, it creates a flexible fluid matrix with which collagen and elastic fibers can bond. HA also ensures the proper activity of cells belonging to the skin’s immune system and creates a protective barrier, preventing, e.g., bacterial penetration [10]. HA is one of the crucial components in numerous biological functions such as angiogenesis, inflammation regulation, and maintaining tissue stability and structure [11,12,13]. Additionally, it also helps in various cellular functions such as cell differentiation, proliferation, and adhesion [15,16]. Hyaluronic acid also plays a crucial role in the healing and re-epithelialization process thanks to the promotion of fibroblast proliferation, migration and adhesion to the wound site, as well as the stimulation of collagen production.

The molecular formula of HA is (C_14_H_21_NO_11_)_n_, and its chemical structure comprises repeating disaccharide units of D-glucuronic acid and N-acetyl-D-glucosamine arranged alternately linked via β-1,4 and β-1,3 glycosidic bonds forming a polymeric structure [15,16].

Under physiological conditions, HA acquires a double helical structure, which confers molecular stability and rigidity. The carboxylate, acetamido, and hydroxyl groups of hyaluronic acid form hydrogen bonds with water molecules, contributing to its strong hydrophilicity. These interactions enhance the water retention ability of HA, contributing to its high hydrophilicity, which is essential for maintaining an appropriate hydration level and preventing the tissues from undergoing physical stresses [17]. The molecular weight of naturally occurring hyaluronic acid in humans ranges from 10^5^ to 10^7^ Da and varies depending on its localization in different tissues. Based on the molecular weight, HA is categorized into high, medium, and low-molecular-weight HA. High molecular weight HA has a molecular weight ≥ 1000 kDa, medium-molecular weight HA has a molecular weight between 250 and 1000 kDa, whereas low-molecular weight HA has a molecular weight between 10 and 250 kDa [18]. It has been reported that the physicochemical properties and biological functions of HA are interlinked with its molecular weight. High-molecular-weight HA generally suppresses angiogenesis, modulates immune responses, and reduces inflammation; it is also subject to enzymatic and thermal degradation under certain conditions [13]. It also plays an important role in several biological processes such as tissue regeneration, and wound healing. Additionally, due to high viscoelasticity, they act as a lubricant in synovial fluid and protect cartilage [19]. A reduction in HMW-HA content or its degradation results in tissue homeostasis disruption. This condition promotes inflammation, affects the loss of viscoelasticity in the joints, and may be associated with the development of pathological processes, i.e., excessive angiogenesis, fibrosis, or cartilage degradation [20]. However, medium and low molecular weight HA triggers angiogenesis, inflammation, and stimulates the immune system. It also promotes the formation of new lymphatic vessels due to the interaction of endothelial lymphatic vessel receptors 1 [13,19]. Moreover, medium and low molecular weight HA exhibits higher stability towards enzymatic and thermal degradation. HA has been proven as a bio-functional, biocompatible, and biodegradable biopolymer owing to its unique chemical structure. It also demonstrates physicochemical properties such as high viscoelasticity and high-water holding capability, making HA a promising candidate for biomedical applications [13,14,15,16,17,18,19].

Chemical modification of HA via a crosslinking strategy, which involves carboxyl, hydroxyl, and N-acetyl groups, can be employed to alter its physical and chemical properties according to the intended application. Crosslinking of HA can be achieved through physical crosslinking, chemical crosslinking, and enzymatic crosslinking [21]. In physical crosslinking, physical stimuli such as temperature, pH, and pressure induce gelation. Chemical crosslinking can be achieved either using a crosslinker to form a 3D structure or by modifying the HA chain with a functional group that can easily undergo crosslinking. Whereas enzymatic crosslinking involves inducing covalent bonding between HA molecules using enzymes [21,22].

The half-life of native hyaluronic acid in the skin is 24–48 h due to enzymatic degradation catalyzed by hyaluronidases. This form of HA also has poor mechanical properties and dissolves easily in an aqueous environment. These properties pose a significant obstacle in the design of HA-based therapies and limit their clinical usefulness. The use of chemically modified HA or its incorporation into hybrid matrices improves its stability, durability, and rheological parameters [23]. However, there is a risk that the cross-linking reagents, catalysts, or reaction initiators used for the chemical modification of HA may be toxic, especially with prolonged exposure [24]. The transfer of chemically modified HA to clinical practice is also delayed due to the insufficient chemical characteristics of bioconjugates, differences between production batches, and the complexity of their production technology [23].

## 3. Growth Factors and Their Importance in Skin Regeneration and Wound Healing Processes

Due to the complexity and importance of skin functions for the functioning of the entire human body, its proper functioning and maintenance of its continuity are essential. Hence, over the years, many mechanisms have evolved to ensure effective skin regeneration and healing of skin defects.

**Figure 1:**
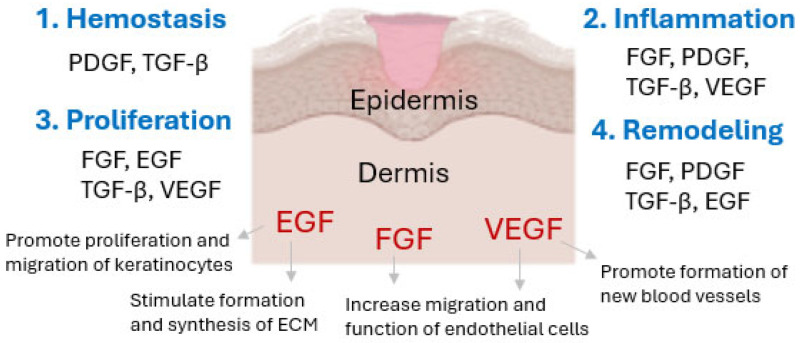
Growth factors involved in skin regeneration and their importance in wound healing (own elaboration based on [31,32]).

The process of skin healing is a dynamic process aimed at restoring homeostasis and functionality of damaged tissue. It consists of four stages, such as coagulation, inflammation, proliferation and tissue maturation. As a result of tissue damage, in the first stage, hemostasis mechanisms are activated, including constriction of blood vessels, followed by aggregation of platelets, which leads to the formation of a clot containing cross-linked fibrin. The inflammatory stage involves the migration of neutrophils and monocytes, phagocytosis of bacteria, and the release of proteolytic enzymes, which are intended to cleanse the wound. The proliferation phase, which involves numerous cells, i.e., fibroblasts, keratinocytes and endothelial cells, includes such main processes as the extracellular matrix (ECM) biosynthesis, epithelialization, and angiogenesis. Remodeling is the final phase of the healing process, during which the ECM rebuilds and granulation tissue matures, forming a scar, which leads to increased mechanical strength of the newly formed tissue [25]. An important step in tissue formation, repair, and the maintenance of good skin condition is the proper proliferation and migration of keratinocytes and fibroblasts, which depend on growth factors (GFs) such as epidermal growth factor (EGF), fibroblast growth factor (FGF), and vascular endothelial growth factor (VEGF) [26,27,28,29,30]. The importance of growth factors in the regeneration and healing process is presented in Figure 1.

GFs are physiologically active proteins that control cell growth, differentiation, and metabolism. They play a key role as signal transducers in most tissue processes, where they are responsible for proliferation, differentiation, chemotaxis, and morphogenesis [26,27]. Growth factors also play an important role in the healing process, which includes stages such as coagulation, inflammation, proliferation, and tissue maturation [27]. An important step in tissue formation, repair, and the maintenance of good skin condition is the proper proliferation and migration of keratinocytes and fibroblasts, which depend on growth factors such as epidermal growth factor (EGF), fibroblast growth factor (FGF), and vascular endothelial growth factor (VEGF) [27,28,29,30].

The epidermal growth factor (EGF) family includes transforming growth factor-α (TGF-α), heparin-binding EGF (HB-EGF), amphiregulin, betacellulin, and epiregulin. EGF is a polypeptide composed of 53 amino acid residues, involved in numerous cellular processes occurring in the skin. EGF exerts its biological activity by binding to its receptor, EGFR (epidermal growth factor receptor), a protein tyrosine kinase receptor, also known as ErbB1 or HER1. EGF plays a key role in the repair processes of damaged structures, thanks to its ability to stimulate the proliferation and differentiation of stem cells, which are essential in the process of tissue reconstruction. EGF stimulates the migration of keratinocytes, as well as fibroblasts and vascular endothelial cells, thus facilitating the formation of granulation tissue and re-epithelialization. Supports angiogenesis, thereby increasing the supply of oxygen and nutrients to regenerating tissue. Furthermore, by influencing the expression of genes related to the biosynthesis of lipids and proteins in the stratum corneum, it plays an important role in building the skin barrier [28,33,34].

Fibroblast growth factors (FGFs) are a large family of cell-signaling proteins produced by different types of cells that act through tyrosine kinase receptors known as FGF receptors (FGFRs) [29,35]. Fibroblast growth factor/FGF receptor (FGF/FGFR) signaling plays a key role in adult homeostasis by regulating differentiation, proliferation, and apoptosis of various cell types. FGFs are active mitogens that stimulate the proliferation of many cells of ecto-, meso-, and endodermal origin. An exception is FGF-7, also known as keratinocyte growth factor (KGF-1), which primarily affects keratinocytes. In addition to their mitogenic effects, FGFs also induce cell migration and proper differentiation, and even play a protective role in conditions of cellular stress. Literature data indicate that FGF factors play a role in tissue repair and regeneration. The interaction between FGF factors and other key signalling molecules is also emphasised. Both acidic FGF (aFGF) and basic FGF (bFGF) influence the wound healing process by participating in angiogenesis [29,35].

The VEGF family comprises four VEGF isoforms (A, B, C, D), which act primarily through the VEGF-R1 and VEGF-R2 receptors. They are among the most potent inducers of angiogenesis (physiological and pathological) and vasculogenesis. They also participate in the development of lymphatic vessels. VEGF participates in all phases of angiogenesis, with its role being particularly crucial during the initiation of new blood vessel formation [30]. It has been demonstrated that, among other angiogenic growth factors, VEGF has a significant impact on various elements of the healing process. Studies have shown that VEGF sends signals that influence collagen deposition and epithelialisation [36].

One strategy to accelerate wound healing involves incorporating GFs into current treatment methods [37]. However, the use of GFs in vivo is associated with certain limitations. These include, among others, the short half-life of growth factors, instability at room temperature, as well as loss of activity after loading onto a matrix [38,39,40]. These limitations in the use of GFs in the context of skin wound healing highlight the need to develop effective delivery systems that increase their efficacy [41]. Despite advances in application methods, ensuring stable and controlled release of growth factors at the wound site remains a challenge. The most frequently studied categories of biomaterials for delivering growth factors to skin wounds include decellularised ECM scaffolds, protein- or polysaccharide-based sponges, as well as hydrogels [41,42].

## 4. Review of Studies on Hyaluronan-Based Hybrid Systems with Growth Factors in the Treatment of Chronic Wounds

The properties of hyaluronic acid make it ideal for biomedical applications. This compound occurs naturally in the body, is biocompatible, biodegradable, and non-immunogenic. Its antioxidant and anti-inflammatory properties have been confirmed. It has the ability to absorb large amounts of water, creating a hydrophilic environment conducive to cell proliferation [43]. Despite its many beneficial properties, its practical clinical application is fraught with certain difficulties. One of the key limitations of HA is its poor mechanical properties. In addition, it degrades rapidly in vivo [44]. One solution to these problems is the development of hybrid systems. Modification of HA by coformulation with another polymer (e.g., alginate, collagen) improves the mechanical properties of the dressing, increases the resistance of the matrix to compressive forces, reduces the rate of degradation, and allows the design of a carrier enabling the controlled release of active substances (API). In the case of complex hybrids, the risk of immune reactions potentially increases, especially when hyaluronic acid is combined with animal-derived collagen or chitosan [45,46]. HA-based hybrid hydrogels allow the formulation of various drug delivery systems, including films, sponges, and beads. HA, because of its unique hygroscopic, rheological, and viscoelastic properties, is an important biomaterial worth considering in the context of wound healing [42]. An important aspect of current research is the evaluation of hyaluronic acid-based wound dressings, films or hydrogels enriched with other therapeutic agents to understand whether the scarring process can be further enhanced [10]. Table 1 provides an overview of hyaluronan-based hybrid systems as carriers of growth factors in the treatment of chronic wounds.

Choi et al. [38] developed a hydrogel matrix based on hyaluronan acid (HA) and collagen (HCD), with the addition of Pluronic F68. The formulation was supplemented with structurally stabilised epidermal growth factor (S-EGF) and structurally stabilised basic fibroblast growth factor (S-bFGF), which were modified to obtain thermostable APIs with a longer half-life compared to standard forms. The presence of disulfide bonds in the protein structure increases their structural stability without altering their affinity for specific receptors. The GF doses used were 0.1, 0.3, 1.0, and 2.5 μg/cm^2^. Cytotoxicity tests confirmed the safety of the preparations. The HCD matrix was an effective carrier of S-EGF and S-bFGF, ensuring their biological stability. The growth factors were released from the matrix gradually over 21 days. The formulations developed were biocompatible, guaranteeing their safety in use. In another study, Choi et al. [47] tested the efficacy of the developed HCD hydrogel with S-EGF (0.3 μg/cm^2^) and S-bFGF (1 μg/cm^2^) in vivo in a diabetic wound model in mice with type I diabetes. Faster tissue healing was observed compared to the control group. Stimulation of re-epithelialisation, neovascularisation, and collagen deposition at the healing site was confirmed. In another study, the same authors [40] prepared a formulation consisting of hyaluronic acid and collagen, into which they incorporated two growth factors, S-EGF and S-bFGF (Dual-HCD; Dual Hyaluronate-Collagen Dressing) in proportions of 1:1, 1:2, and 2:1. The authors found no cytotoxicity of the developed system towards fibroblast cells (BALB/3T3, NIH/3T3). Dual-HCD (S-EGF: S-bFGF = 1:2) increased cell proliferation, collagen deposition, and supported re-epithelialisation processes. The release of growth factors from the Dual-HCD matrix occurred over a prolonged period of 3 days (Q = 97%). The efficacy of Dual-HCD + S-EGF and S-bFGF (1:2) was tested in vivo in a diabetic wound model in mice with type II diabetes. Better wound healing was observed after 14 days compared to the matrix containing a single growth factor or the HCD matrix alone. Complete wound closure was observed after 21 days. S-EGF and S-bFGF (in an optimal ratio of 1:2) showed synergistic effects in stimulating angiogenesis and promoted faster epithelialisation (2.7-fold increase vs. control groups). Histopathological examination revealed collagen deposition at a level of 76.5% and increased chemotactic migration. The authors recommend conducting clinical trials to further evaluate the developed preparation.

Kondo et al. [42] designed a dressing consisting of hyaluronic acid (a mixture of high molecular weight HA/HMW-HA and low molecular weight HA/LMW-HA) and collagen sponge (Col) containing epidermal growth factor (EGF, 2 μg/cm^2^). The resulting spongy dressing was evaluated in vitro and in vivo. It was found that HA-Col-EGF stimulated cell proliferation after UV irradiation and after sterilisation at 110 °C for 1 h. EGF retained its activity under the conditions of preparation sterilisation. Type-II dressing (with EGF) significantly increased the secretion of VEGF (vascular endothelial growth factor) and HGF (hepatocyte growth factor) by fibroblasts compared to Type-I (without EGF). In an in vivo study in a rat postoperative wound model (Sprague-Dawley strain), it was confirmed that after 1 and 2 weeks of therapy with Type-II dressing (with EGF), there was a significant acceleration of wound healing (*p* < 0.01), re-epithelialisation, and granulation tissue formation in the course of angiogenesis (vs. Type-I (without EGF). Type-II dressing (with EGF) also significantly promoted healing (*p* < 0.01) in a rat burn wound model. The authors suggest that the developed dressing may be effective in healing wounds without scarring. The same team, in another study [48], evaluated the effectiveness of Type-II dressing with EGF (HMW-HA + LMW-HA + Col + EGF) in the treatment of diabetic wounds in a mouse model with induced type II diabetes (BKS.Cg- + Lepr^db^/ + Lepr^db^ (db/db)) model). The control group consisted of mice treated with artificial skin (TERUDERMIS^®^). The upper part of the dressings was secured with polyurethane film (Bioclusive^®^). Therapy with Type-II dressing (with EGF) resulted in a significant reduction in wound size compared to artificial skin (*p* < 0.01). The wound size decreased by 80%, then by 15% (compared to the initial state) after 1 week and 2 weeks of use. Greater angiogenesis and granulation tissue formation were observed compared to the control group. Type-II dressing (with EGF) was particularly conducive to the re-epithelialisation process. The authors suggest that the HA-Col-EGF system stimulates cell migration and proliferation processes and may be useful in preparing the wound bed for skin autografting.

Yu et al. [49] verified the therapeutic efficacy of a collagen (Col)- and hyaluronic acid (HA)-based dressing containing EGF or bFGF. It was found that the HA/Col + EGF dressing increased VEGF secretion (a 3.6-fold increase compared to the control sample without growth factor, *p* < 0.01) and HGF (4.6-fold increase compared to the control sample without growth factor, *p* < 0.01) in an in vitro wound model. In turn, the HA/Col + bFGF dressing increased VEGF levels by as much as 10.2-fold and HGF levels by 6.3-fold compared to the control sample without growth factor (*p* < 0.01). Each of the two analysed preparations stimulated fibroblasts to release increased amounts of VEGF and HGF, which (acting synergistically) can induce angiogenesis. Thanks to the promising results of the study, the authors undertook clinical trials [50]. The therapeutic efficacy of the HA/Col + EGF dressing was tested in a group of patients with burn wounds and ulcerative skin lesions. Sixteen patients aged 59 ± 20 years participated in the study. The dressing was applied at intervals of 3 to 5 days for a period of at least 6 weeks. The results confirmed the efficacy and safety of the dressing in all cases. The preparation accelerated the process of granulation and epithelialisation, significantly reducing the size of the wound. The authors recommend the HA/Col + EGF dressing for the treatment of difficult-to-heal wounds.

The team of Matsumoto et al. [51] confirmed the therapeutic efficacy of the HA/Col + Arg + EGF dressing in a model of fibroblasts taken from a fragment of human skin and in a preclinical study in a rat (Sprague-Dawley) burn wound model. It was found that EGF accelerates re-epithelialisation and induces neutrophil infiltration in granulation tissue 1 week after application. The stimulation of re-epithelialisation by EGF was confirmed in a preclinical study. EGF caused moderate inflammation, suggesting that cytokines derived from inflammatory cells influence the wound healing process.

Niiyama et al. [52] investigated the potential of HA/Col + VC + EGF dressing in a fibroblast model (cultured dermal substitute; CDS) and in a mouse model of genetically determined type II diabetes (BKS.Cg– + Lepr^db^/ + Lepr^db^ (db/db). It was found that fibroblasts in CDS covered with a layer of HA/Col + EGF dressing released 3.6 times more VEGF and 3.0 times more HGF compared to the HA/Col control group (*p* < 0.01 and *p* < 0.05, respectively). In turn, the use of the HA/Col + VC + EGF dressing resulted in the release of 4.2 times more VEGF and 6.0 times more HGF compared to the control group (*p* < 0.01). The study lasted 7 days. Studies conducted in a mouse model confirmed that the extent of granulation tissue formation after 1 and 2 weeks of applying HA/Col + VC + EGF to the wound was significantly greater compared to the control group (1 week: *p* < 0.05, 2 weeks: *p* < 0.01). This study group also showed increased angiogenesis and collagen increase within the wound.

Sawa et al. [33] prepared an allogeneic skin substitute (CDS) based on collagen-hyaluronic acid, with incorporated EGF. The matrix contained human dermal fibroblasts, which showed the ability to secrete proangiogenic cytokines VEGF and HGF. The use of the CDS-EGF dressing resulted in the release of 3.2 times more VEGF and 7.9 times more HGF compared to the CDS control group (*p* < 0.001). Mineo et al. [53] confirmed the effectiveness of CDS-EGF in producing increased amounts of VEGF and HGF. The authors examined the therapeutic potential of the developed dressing in a model of a deep burn wound in Sprague Dawley rats. Artificial skin was applied to the experimentally induced wound for 7 days, and then its ability to form a wound bed was assessed. It was found that CDS-EGF promotes angiogenesis in the early stages of healing (within 3 days) and inhibits the inflammatory response (vs. the control group). In a subsequent test, an autologous skin graft procedure was performed on rats on the wound bed. The dermal substitute promoted tissue regeneration and the formation of a vascularised bed, which facilitated graft implantation. Kuroyanagi et al. [54] used a collagen-hyaluronic acid matrix to prepare a dermal substitute (CDS). Its upper layer consisted of an HA-Col patch incorporating EGF, while the lower layer consisted of a spongy HA-Col matrix with fibroblasts. The EGF contained in the dressing significantly stimulated fibroblasts to release VEGF and HGF (vs. EGF-free CDS, *p* < 0.01). Iijima et al. [55] confirmed in the same research model that CDS-EGF stimulates fibroblasts to synthesise increased amounts of VEGF and HGF in a dose-dependent manner. The authors confirmed that the optimal concentration of EGF in the matrix is between 0.1 and 0.2 µg/cm^2^. Higher concentrations of EGF, on the other hand, inhibit fibroblast function. Membranes containing EGF at a concentration of 0.2 µg/cm^2^ stimulated fibroblasts in CDS to release 2.3 times more VEGF and 5.8 times more HGF compared to the control group (*p* < 0.01).

Thönes et al. [56] developed a biocompatible hybrid hydrogel based on hyaluronic acid (HA) and type I collagen, modified with sulphated derivatives sHA for use in wound treatment. Heparin-binding EGF-like growth factor (HB-EGF) was incorporated into the resulting matrix. It was found that the hydrogel stabilises the structure of HB-EGF and maintains its bioactivity for at least 72 h. The release of HB-EGF from the polymer carrier in vitro was prolonged. HB-EGF was released from the sHA-containing hydrogel within 24 h (the study lasted 72 h). In addition, it stimulated the migration of keratinocytes (HaCat) in a dose-dependent manner (10 ng/mL, *p* < 0.01). The inclusion of sHA in the hydrogel structure improved the effectiveness of the preparation in stimulating keratinocyte proliferation and migration. The HA/collagen + sHA + HB-EGF formulation (approx. 1–10 ng/mL) accelerated wound healing more effectively than the HB-EGF solution alone (400 ng/wound), indicating that the developed matrix improves the bioavailability and stability of the growth factor in the skin.

Wang et al. [57] designed a matrix (CHS-PDA-2@EGF) based on collagen and hyaluronic acid (CHS) modified with polydopamine (PDA) at concentrations of 0.5 and 1.2 mg/mL, to which they added epidermal growth factor (EGF). The carrier had the ability to swell and exhibited a coagulation effect with moderate degradation. Preformulation studies confirmed its biocompatibility and lack of cytotoxic effect using RAW264.7 and NIH3T3 cell lines. EGF was released from the carrier in a sustained manner. The efficacy of the developed preparation was analysed in vivo in a rat model with induced type II diabetes. It was found that the formulation exhibits antioxidant and anti-inflammatory properties and may inhibit M1 macrophage polarisation. CHS-PDA with the addition of EGF stimulated the healing of diabetic wounds and improved their closure compared to the control groups (*p* < 0.05). A significant effect of EGF on the stimulation of cell proliferation in the wound was found.

The team of Liu et al. [58] prepared a matrix based on hyaluronic acid (HA) and sodium alginate (ALG) with varying compositions (from 1:1 to 1:5), into which they incorporated recombinant human epidermal growth factor (rh-EGF). rh-EGF was released from the hydrogel in vitro over a period of 4 days (Q = 90%). It was found that ALG-HA/rh-EGF improved the proliferation and adhesion of human L929 cells. The hydrogel with EGF showed biocompatibility. In the cytotoxicity test (MTT), the hydrogels were not toxic to mouse L929 fibroblasts. In blood compatibility studies (platelet adhesion test), the hydrogel showed compatibility comparable to polyurethane (PU, Pellethane^®^, Lubrizol Advanced Materials, Ritterhude, Germany), suggesting that ALG-HA/rh-EGF does not cause unacceptable platelet activation. Ali et al. [59] developed an alginate and hyaluronic acid (ALG–HA, 80:20 ratio) bead system containing vascular endothelial growth factor (VEGF). In vitro studies showed that VEGF was released from the ALG-HA/VEGF preparation within 2 days (Q = 92%) and from the ALG-HA-Hep/VEGF preparation within 5 days (Q = 85%). Heparin incorporated into the polymer matrix showed a strong affinity for VEGF. The ALG-HA/VEGF preparation showed biocompatibility with calf pulmonary artery endothelial cells (CPAE) and increased expression of VCAM1 (vascular cell adhesion proteins) and eNOS (endothelial enzyme). The therapeutic efficacy of the preparation was determined in an in vivo study using a rat (Sprague-Dawley) wound model. In the second week of treatment, the wound covered with ALG-HA/VEGF closed in 70% of cases vs. 50% in the control group (*p* < 0.01). The dressing stimulated vascularisation and the production of collagen type-1 (Col-1) and fibronectin (FN). The same authors, in a subsequent study [60], replaced VEGF with EGF in the developed ALG80: HA20 polymer beads. In an in vitro study, the authors confirmed that the developed preparation was biocompatible with L929 fibroblasts. It was observed that EGF was released from the ALG-HA/EGF100 preparation within 2 days (Q = 85%), while 50% of the initial dose was released from the ALG-HA-Hep/EGF100 preparation within 2 days. Heparin incorporated into the polymer matrix also showed a strong affinity for EGF. Both ALG-HA/EGF100 and ALG-HA/EGF150 preparations showed highly significant expression of FLK-1 (Fetal Liver Kinase, one of the VEGF receptors) and ICAM-1 (Intercellular Adhesion Molecule-1) in rbMSC (rat bone mesenchymal stem cells). In a subsequent in vivo study, two preparations (ALG-HA/EGF100 and ALG-HA/EGF150) were applied to a wound in a rat model. After 14 days of observation, a reduction in wound area of 69% and 77% (compared to the control group, *p* < 0.001) was observed, confirming the therapeutic efficacy of the developed dressings.

### 4.1. Mechanisms Responsible for the Beneficial Effects of Hybrid Systems Containing Growth Factors

The developed hybrid hydrogels support the wound healing process through the synergistic action of their components. Collagen/hyaluronic acid and alginate/hyaluronic acid systems are biocompatible and biodegradable matrices that provide a moist wound environment, which promotes cell migration and proliferation [57]. Hyaluronic acid reduces inflammation and acts as a factor supporting cell migration and angiogenesis [42]. High molecular weight HA (HMW-HA) ensures adequate moisture in the wound, which affects the stability and therapeutic activity of EGF released from the hybrid system [42]. Low molecular weight HA (LMW-HA) promotes angiogenesis, supporting the formation of new blood vessels within the wound [50], and stimulates granulation tissue formation [48]. Collagen acts as an effective chemoattractant for fibroblasts, supporting their migration and participation in granulation tissue formation [50]. Alginate has antibacterial properties [59].

Wound healing is the result of the interaction of many molecular factors. EGF binds to EGFR receptors on the cell surface, activating MAPK and PI3K/Akt signalling pathways. These, in turn, regulate cell proliferation, migration, differentiation, and survival, increase collagen synthesis, and accelerate re-epithelialisation. MAPK activation affects the expression of genes related to the cell cycle, increasing cell proliferation at the wound site, participating in tissue reconstruction, and accelerating the healing process [59,60]. Hydrogels containing epidermal growth factor (EGF) indirectly stimulate fibroblasts to increase the production of angiogenic factors (VEGF and HGF). The secretion of these cytokines promotes the formation of blood vessels, oxygenating and nourishing diseased tissues. A hybrid hydrogel consisting of hyaluronic acid (HA), collagen (Col) or alginate + epidermal growth factor (EGF) vs. EGF alone more effectively promotes angiogenesis and collagen deposition, which results in faster granulation tissue formation and epithelialisation. The mechanism of action of the system is based on the activation of fibroblasts by EGF, increased secretion of VEGF and HGF growth factors, and the induction of repair processes at the wound site [42,52]. In the case of Dual-HCD hydrogels (S-EGF + S-bFGF), S-EGF stimulates the proliferation and migration of keratinocytes, which accelerates re-epithelialisation. S-bFGF, on the other hand, supports angiogenesis by stimulating the proliferation and migration of vascular endothelial cells. The effect of these actions indirectly leads to increased production of VEGF and HGF cytokines, which support the formation of granulation tissue [40].

### 4.2. Significance and Limitations of Research Based on Animal Models

61% of the studies included in the review were conducted using animal models. The rodent excision wound healing model is a well-established and widely used approach in preclinical research. Studies dating back to the late 1950s suggest that the limitation of this model stems from differences in the wound healing mechanism in rodents vs. humans. It has been found that wounds in rodents heal by contraction (which accounts for approximately 80% of the wound healing process), while in humans they heal by re-epithelialisation and granulation tissue formation [61]. Rodent skin has a panniculus carnosus layer, which is responsible for rapid wound contraction [62]. Studies conducted by Chen et al. [61] confirm, however, that simple excised wounds in rodents heal by contraction and re-epithelialisation, and the model may be useful in the analysis of early healing. In turn, the use of a mouse wound model with a stiffening splint can prevent contraction, and the wound will heal through re-epithelialisation and granulation tissue formation [63]. Burn wounds in rodent models are induced by skin burns with water or thermal damage. They can be used to evaluate re-epithelialisation, granulation tissue formation, angiogenesis, contraction, and scarring [62].

Rodent models of diabetic wounds, despite their usefulness in studying the therapeutic efficacy of developed preparations, have significant limitations. Models based on induced diabetes, metabolic disorders, and the use of modified db/db and ob/ob mice with type 2 diabetes limit the extrapolation of results to clinical pathologies observed in humans. Streptozotocin-induced diabetes does not fully reflect the complex nature of diabetes in patients, while the genetic model differs in terms of the dynamics of diabetes development, immune response, and vascular processes. It should be noted that models of impaired acute wound healing can provide information on delayed healing, but do not fully reflect the nature of chronic wounds observed in humans [64,65].

Animal models allow the therapeutic efficacy of preparations in chronic wound healing to be assessed, but they do not fully reflect the wounds of patients in clinical settings.

## 5. Materials and Methods

### 5.1. Focused Questions

Are hyaluronan-based hybrid systems as growth factor carriers effective wound dressings?

### 5.2. Eligibility Criteria

The Google Scholar, Web of Science and Medline (PubMed) databases were searched using a combination of MeSH terms: “hyaluronic acid”, “hyaluronan”, “growth factor”, “hydrogel”, “hybrid hydrogel”, “polymer”, “polymeric materials”, “hybrid systems”, “wounds”, “wound healing”, “chronic wounds”, “wound dressings”, “hybrid wound dressings”, “biopolymers”, “biomaterials”. Two authors independently searched the databases. The review included articles published in peer-reviewed journals that contained the specified keywords in their titles and/or abstracts. Studies published up to October 2025 were included, with particular emphasis on publications from the last 25 years. Inclusion criteria were as follows: original works containing data from basic, preclinical, and clinical studies, with a positive opinion from the bioethics committee. Hybrid systems were included whose components were hyaluronic acid/hyaluronan and another polymer (e.g., collagen, alginate) with an incorporated growth factor. Exclusion criteria were as follows: articles that studied hyaluronan-based hybrid carriers without growth factors and those concerning applications other than the treatment of chronic wounds. Letters to the editor, non-scientific publications, studies published on websites, and in newsletters were not included in the review.

## 6. Conclusions

Studies confirm the potential of hyaluronan-based hybrid systems containing epidermal growth factor (EGF), vascular endothelial growth factor (VEGF), and fibroblast growth factor (FGF) as a wound dressing. Hybrid systems have been developed that enable the sustained release of growth factors and enhance the effectiveness of wound closure and healing. They ensure GF stability and protect them from enzymatic degradation. They promote tissue regeneration, e.g., by stimulating fibroblast proliferation and angiogenesis. Although these advanced material systems possess key properties that make them ideal for biomedical applications, further research is needed to optimize their mechanical strength, degradation/release kinetics, and growth factor concentration. Research is also needed to determine the long-term safety of HA-based hydrogel systems. Nevertheless, such dressings have shown good application prospects and are a promising tool in the treatment of chronic wounds. In the future, clinical trials should be conducted to confirm the observed effects and investigate the mechanisms of action.

## Figures and Tables

**Table 1:** Review of hyaluronan-based hybrid systems as growth factor carriers in the treatment of chronic wounds.

Author, Year	Hybrid Matrix/Preparation Method	Growth Factor(s)	Research Model	Therapeutic Efficacy
Choi S.M., 2016 [38]	hyaluronate–collagen dressing (HCD) Mw HA: n.d. Physical blending	EGF bFGF (S-EGF, S-bFGF: 0.1, 0.3, 1.0, 2.5 μg/cm^2^).	in vitro	extended release; no toxic effects on cells observed; no inflammatory response occurred
Choi S.M., 2018 [47]	hyaluronate–collagen dressing (HCD) Mw HA: n.d. Physical blending	EGF bFGF (S-EGF: 0.3 μg/cm^2^; S-bFGF: 1 μg/cm^2^)	in vitro/in vivo type I and II diabetic mouse models	accelerated wound healing through stimulation of re-epithelialisation, neovascularisation, and collagen deposition
Kim M.S., 2021 [40]	hyaluronate–collagen dressing (HCD) Mw HA: n.d. Physical blending	EGF bFGF (S-EGF: S-bFGF, 1:2, 1 μg/cm^2^)	in vitro/in vivo type I diabetic mouse models	Dual-HCD exhibits a synergistic effect; the ability to accelerate diabetic wound healing; induction of re-epithelialisation, neovascularisation, and collagen deposition; stimulation of HaCaT cell migration (in vitro); therapeutic efficacy in the treatment of chronic diabetic wounds.
Kondo S., 2012 A [42]	collagen–hyaluronic acid sponge polydisperse: HMW-HA 2000 kDa/LMW-HA (autoclave at 120 °C for 1 h): n.d. Physical blending + freeze-drying + UV crosslinking of collagen	EGF (2 μg/cm^2^)	in vitro/in vivo Sprague–Dawley rat, surgical wound; Sprague–Dawley rat, burn wound	reduction in wound size; re-epithelialisation and formation of granulation tissue associated with angiogenesis; increased secretion of VEGF and HGF by fibroblasts
Kondo S., 2012 B [48]	collagen–hyaluronic acid sponge polydisperse: HMW-HA 2000 kDa/LMW-HA (autoclave at 120 °C for 1 h): n.d. Physical blending + freeze-drying + UV crosslinking of collagen	EGF (2 μg/cm^2^)	in vivo type II diabetic db/db mice, burn wound	positive effect on burn wound healing; reduction in wound surface area; angiogenesis, re-epithelialisation, and granulation tissue formation
Yu A., 2013 [49]	collagen–hyaluronic acid sponge polydisperse: HMW-HA 2000 kDa/LMW-HA (autoclave at 120 °C for 1 h): n.d. Physical blending + freeze-drying + UV crosslinking of collagen	EGF bFGF (1 μg/cm^2^)	in vitro	increased secretion of VEGF and HGF; modulation of fibroblast response and promotion of angiogenesis
Yu A., 2015 [50]	collagen–hyaluronic acid sponge polydisperse: HMW-HA 2000 kDa/LMW-HA (autoclave at 120 °C for 1 h): n.d. Physical blending + freeze-drying + UV crosslinking of collagen	EGF (1 μg/cm^2^)	clinical trial human patients with burns, chronic ulcers, and traumatic skin defects	confirmed therapeutic efficacy of the formulation; granulation tissue formation and rapid re-epithelialisation within the wound
Matsumoto Y., 2010 [51]	collagen–hyaluronic acid sponge containing arginine (Arg) polydisperse: HMW-HA: n.d./LMW-HA (autoclave at 120 °C for 1 h): n.d. Freeze-drying of HMW-HA with EX 810 (chemical crosslinking), blending with autoclaved LMW-HA containing Arg; immersion, incubation at 4 °C, freezing	EGF: 100 μg Arg: 0.5 g	in vitro/in vivo Sprague–Dawley rat, surgical wound; Sprague–Dawley rat, burn wound	accelerated re-epithelialisation; inflammatory response beneficial to healing processes
Niiyama H., 2014 [52]	collagen–hyaluronic acid sponge containing vitamin C derivative (VC) polydisperse: HMW-HA: n.d./LMW-HA (autoclave at 120 °C for 1 h): n.d. HMW-HA and LMW-HA blended with heat-denatured collagen; poured into trays, refrigerated 4 °C, frozen at −85 °C, freeze-dried; UV irradiation of collagen for crosslinking	EGF (1 μg/cm^2^) VC (2.5 mg/cm^2^)	in vitro/in vivo type II diabetic db/db mice, dorsal wound	in vitro production of VEGF and HGF; stimulation of granulation tissue formation associated with angiogenesis and collagen deposition in vivo
Sawa M., 2013 [33]	collagen–hyaluronic acid sponge polydisperse: HMW-HA 2000 kDa/LMW-HA (autoclave at 120 °C for 1 h): n.d. Physical blending + freeze-drying + UV crosslinking of collagen	EGF (1 μg/cm^2^)	in vitro	stimulation of fibroblasts to release VEGF and HGF
Mineo A., 2013 [53]	collagen–hyaluronic acid sponge polydisperse: HMW-HA 2000 kDa/LMW-HA (autoclave at 120 °C for 1 h): n.d. Physical blending + freeze-drying + UV crosslinking of collagen	EGF (1 μg/cm^2^)	in vitro/in vivo Sprague–Dawley rat, burn wound	stimulation of fibroblasts to release VEGF and HGF; promotion of angiogenesis, and formation of a vascularised wound bed
Kuroyanagi M., 2014 [54]	collagen–hyaluronic acid sponge polydisperse: HMW-HA 2000 kDa/LMW-HA (autoclave at 120 °C for 1 h): n.d. Physical blending + freeze-drying + UV crosslinking of collagen	EGF (1 μg/cm^2^)	in vitro	stimulation of fibroblasts to release VEGF and HGF
Iijima E., 2014 [55]	collagen–hyaluronic acid sponge polydisperse: HMW-HA 2000 kDa/partially hydrolyzed LMW-HA 150 kDa Physical blending + freeze-drying + UV crosslinking (collagen or EX810)	EGF (0.1, 0.2, 0.5 μg/cm^2^)	in vitro	dose-dependent stimulation of fibroblasts to release VEGF and HGF
Thönes S., 2019 [56]	hyaluronate–collagen dressing supplemented with acrylated sulfated hyaluronan (sHA); native HA 1100 kDa; LMW-HA and sulfated oligosaccharides–depending on degree of polymerization (dp4, dp6) Physical blending + freeze-drying + UV crosslinking of collagen	heparin-binding EGF-like growth factor (HB-EGF) (10 µg/mL)	in vitro	ensuring the bioactivity of the growth factor; stimulation of keratinocytes and fibroblasts; effective wound healing
Wang Y., 2022 [57]	collagen–hyaluronic acid composite HA: 150–250 kDa Physical blending + freeze-drying + EDC/NHS crosslinking	EGF	in vitro/in vivo type II diabetic Sprague-Dawley rats	confirmed healing efficacy in chronic diabetic wounds; prolonged release; combination of antioxidant properties and inflammation modulation
Liu Y., 2014 [58]	alginate–hyaluronic acid (from 1:1 to 1:5,) hydrogel Mw HA: n.d. Physical blending + chemical crosslinking (using ADH, EDC, and Ca^2+^)	rh-EGF (100.0 μg/mL)	in vitro	extended release, biocompatible, non-toxic
Ali M., 2023 A [59]	alginate–hyaluronic acid (80:20) composite beads, heparin crosslink Mw HA: n.d. Physical blending + ionic crosslinking with Ca^2+^	VEGF (150 ng/mL) Heparin (Hep) 5IU	in vitro/in vivo Sprague-Dawley rats, full-thickness skin wounds	extended release; 70% wound closure two weeks after implantation, stimulation of vascularisation, production of collagen type-1 (Col-1) and fibronectin (FN)
Ali M., 2023 B [60]	alginate–hyaluronic acid (80:20) composite beads, heparin crosslink Mw HA: n.d. Physical blending + ionic crosslinking with Ca^2+^	EGF (100.0, 150.0 ng/mL) Heparin (Hep) 5IU	in vitro/in vivo Sprague-Dawley rats, full-thickness skin wounds	prolonged release; high expression of FLK-1 and ICAM-1 in rbMSC, 69% and 77% reduction in wound area

Abbreviations: ADH, adipic dihydrazide; db/db—genetically type II diabetic BKS.Cg- + Lepr^db^/ + Lepr^db^ mice; dp, degree of polymerization; EDC-HCl, 1-(3-Dimethylaminopropyl)-3-ethylcarbodiimide hydrochloride; EGF, Epidermal Growth Factor; EX 810, ethylene glycol diglycidyl ether; FGF, Fibroblast Growth Factor; FLK-1, Fetal Liver Kinase 1; HaCaT Cells; Human Adult Low-Calcium High-Temperature Keratinocytes; HGF, hepatocyte growth factor; ICAM-1, Intercellular Adhesion Molecule-1; Mw—weight-average molecular weight; n.d.—not determined; sulfo-NHS, N-Hydroxysulfosuccinimide sodium salt; rbMSCs, rat bone Mesenchymal stem cells; VEGF, vascular endothelial growth factor.

## Data Availability

No new data were created or analyzed in this study. Data sharing is not applicable to this article.
